# Rheumatism: Its Nature, Its Pathology, and Its Successful Treatment

**Published:** 1897-03

**Authors:** 


**Rheumatism: its Nature, its Pathology, and its Successful Treatment.** By T. J. Maclagan, M.D. Second Edition. Pp. xiii., 324. London: Adam and Charles Black. 1896.

What more authoritative can there be than the views of Dr. Maclagan on rheumatism? When one thinks of what rheumatism was in the old days and what it is now, one can but wonder how it was that so many years were required to abolish rheumatic fever as we used to know it. The discussions on salicylates are now over: all the world accepts the fact that the salicyl compounds act like a charm, and are so trustworthy that there is no longer anything to discuss except the theoretical views as to their mode of action.

In this volume the miasmatic theory is expounded, and an explanation is offered of the manner in which the salicyl compounds produce the marked anti-rheumatic effects which they are now acknowledged to possess. The miasmatic theory —which regards the rheumatic poison as a parasitic organism, requiring for its development and action a second factor or nidus which is localised in fibrous and serous tissues, which exists in varying amount in different parts of those tissues, and which, like the nidus of ordinary malarial fevers, may be exhausted and renewed again—satisfactorily explains the leading phenomena of the disease, and the therapeutic test gives the most powerful confirmatory evidence in favour of this view.

